# The impact of atmospheric oxidation on hygroscopicity and cloud droplet activation of inorganic sea spray aerosol

**DOI:** 10.1038/s41598-021-89346-6

**Published:** 2021-05-11

**Authors:** Bernadette Rosati, Sigurd Christiansen, Anders Dinesen, Pontus Roldin, Andreas Massling, E. Douglas Nilsson, Merete Bilde

**Affiliations:** 1 Department of Chemistry, Aarhus University, 8000 Aarhus C, Denmark; 2 Division of Nuclear Physics, Lund University, 22100 Lund, Sweden; 3 Department of Environmental Science, University of Aarhus, 4000 Roskilde, Denmark; 4 Department of Environmental Science, Stockholm University, 11418 Stockholm, Sweden

**Keywords:** Atmospheric science, Atmospheric chemistry, Thermodynamics

## Abstract

Sea spray aerosol (SSA) contributes significantly to natural aerosol particle concentrations globally, in marine areas even dominantly. The potential changes of the omnipresent inorganic fraction of SSA due to atmospheric ageing is largely unexplored. In the atmosphere, SSA may exist as aqueous phase solution droplets or as dried solid or amorphous particles. We demonstrate that ageing of liquid NaCl and artificial sea salt aerosol by exposure to ozone and UV light leads to a substantial decrease in hygroscopicity and cloud activation potential of the dried particles of the same size. The results point towards surface reactions on the liquid aerosols that are more crucial for small particles and the formation of salt structures with water bound within the dried aerosols, termed hydrates. Our findings suggest an increased formation of hydrate forming salts during ageing and the presence of hydrates in dried SSA. Field observations indicate a reduced hygroscopic growth factor of sub-micrometre SSA in the marine atmosphere compared to fresh laboratory generated NaCl or sea salt of the same dry size, which is typically attributed to organic matter or sulphates. Aged inorganic sea salt offers an additional explanation for such a measured reduced hygroscopic growth factor and cloud activation potential.

## Introduction

The oceans contribute the largest constant mass of natural aerosol particles to the atmosphere^1^ in the form of sea spray aerosol (SSA). SSA is produced due to stress applied by winds on the ocean surface, which causes waves to form and break. In this process, air bubbles are entrained in ocean water. When entrained air bubbles rise to the surface they burst, and sea spray droplets are released to the atmosphere. Super-micrometer SSA dominate the emissions by mass but the largest number of SSA is produced in the sub-micrometer size range peaking at dry diameters near 100 nm^2–8^, which implies the presence of large numbers of particles potentially relevant for cloud formation. The chemical composition of SSA is complex and reflects to some extent the composition of the seawater from which it originates^9^ although enrichment of inorganic^10^ as well as organic constituents^11^ have been reported. While several studies have shown that the organic fraction in SSA is variable depending on location and time of the year^12–14^, the inorganic fraction is ever-present and a key ingredient across all size ranges^13^. The hygroscopicity of sea salt has in many years been approximated with that of NaCl but as shown by King et al.^15^ the CCN forming ability of sea salt particles differs from that of NaCl and e.g. Zieger et al.^16^ showed that the hygroscopic growth of sea salt is reduced compared to that of NaCl. Rasmussen et al.^17^ show and Zieger et al.^16^ conclude that the difference in hygroscopicity for inorganic sea salt and NaCl is likely due to hydrates.

Hydrate forming salts contain water molecules within their structures even after extensive drying and thus fundamentally impact water uptake. $\hbox {MgCl}_2$ and $\hbox {CaCl}_2$ are examples of hydrate forming salts present in sea salt aerosol. A recent study by Rosati et al.^18^ shows that aerosolized $\hbox {MgCl}_2$ and $\hbox {CaCl}_2$ particles exhibit a substantially reduced water uptake potential compared to predicted values assuming anhydrous salts. Experimental and predicted values could be reconciled when taking into account the hydration states of the dried particles. Thus, measurements of sea salt water uptake at sub- and supersaturated water vapour conditions are profoundly affected by hydrate forming salts. Their presence in dried particles leads to a reduced water uptake which could at least in part be falsely ascribed to other causes, such as the presence of organic matter.

The role of SSA in aerosol-radiation and aerosol-cloud interactions is strongly dictated by its ability to take up water at both sub- and supersaturated water vapour conditions^19^. Water uptake at sub-saturated conditions, termed hygroscopicity, directly impacts the aerosol optical properties by altering the scattering and absorption potential which is coupled to the ambient relative humidity (RH). Hygroscopicity also influences the SSA’s ability to act as cloud condensation nuclei (CCN) at supersaturated conditions^20^. The atmospheric lifetime of SSA varies between minutes for the largest particles to weeks for accumulation mode particles^1^. During its atmospheric transit, SSA is subjected to so-called ageing processes, which are of physico-chemical nature taking place inside the particles as well as at the surface via interactions with other particles, gases or radiation. Ageing may lead to profound changes in aerosol radiation interactions, and the ability to form cloud droplets. At present, accurate representation of SSA in climate models is hindered by incomplete understanding of the physical and chemical properties of SSA leading to large disagreement between model outputs^21^, where the water uptake is particularly afflicted with large uncertainties^22^.

Laboratory studies have so far primarily focused on the physical and chemical properties of freshly emitted SSA or its major component, NaCl, while field experiments have by necessity mostly included aged SSA^5,7,8,10,15,16,23–31^. The hygroscopic growth factor (GF; defined as the ratio of the wet and dry diameter D; GF(RH) = D_wet_/D_dry_), provides a scale for quantifying hygroscopicity. Based on the hygroscopic growth factor particles are often divided into sub-categories: nearly hydrophobic particles (NHP), less-hygroscopic particles (LHP), more-hygroscopic particles (MHP) and the even more hygroscopic sea-salt particles (SSP)^32^. As pointed out by Swietlicki et al.^32^ the MHP, which are significantly less hygroscopic than pure NaCl particles, have been found to be ubiquitous in the marine environment whereas observation of externally mixed sea salt particles with a distinct SSP–group are limited to high wind speeds. This was also highlighted by the first successful eddy covariance flux measurements over the sea, demonstrating a clear GF dependence on wind speed^33^. Several propositions exist to explain the nature of the reduced hygroscopicity of particles in the marine environment: sea spray may not be present in large enough numbers over the oceans, the particle distribution may be dominated by sulphate containing particles resulting from dimethyl sulphate oxidation, and particles may contain organic species as a result of atmospheric ageing or combinations of these options^32^. An enrichment in organic matter for particles with diameters smaller than 1 $\mu$m has been proposed in several studies to cause a reduction in hygroscopicity^11,26,34–40^, although results from different studies vary quite substantially^7,41^.

Studies dedicated to effects of ageing processes of SSA are scarce. Oum et al.^42^ photolysed ozone ($\hbox {O}_3$) in the presence of aqueous pure NaCl particles, finding that this results in the formation of molecular chlorine ($\hbox {Cl}_{{2}}$). They suggest that this occurs due to generation of hydroxyl radicals (OH) in the aqueous phase which in turn oxidise $\hbox {Cl}^{-}$ finally leading to the release of $\hbox {Cl}_{{2}}$ (see reactions 1–15 in^42^). More recent work has shown that also small amounts of photosensitizers, that are abundant in the marine environment^43^, could cause the release of dihalogens^44^. Another study revealed that the key reactions are dominant at the air–water interface^45^. These studies focused on the gas-phase products of such reactions, neglecting the effects on SSA itself. The experiments by Oum et al.^42^ were further repeated with pure NaCl, investigating changes in the chemical composition of the aerosols^46^. For particles with diameters below 1 $\mu$m it was found that such a reaction gradually depletes the amount of $\hbox {Cl}^{-}$ and consequently replaces it by oxygen (O), hence leading to the formation of NaOH inside the liquid particles^46^. Effects on the physical properties or the use of a complex salt mixture were not investigated.

The current study was designed to explore how ageing by exposure to ozone and UV light affects the hygroscopicity and cloud activation potential of inorganic sea spray aerosol. Through a series of experiments in a well-controlled laboratory environment we examined the hygroscopicity and cloud activation potential of NaCl and artificial sea salt particles of different particle sizes. Additionally, the volatility of the particles was investigated and microscopy analysis was performed to yield insight into the elemental composition of fresh and aged particles.

## Results and discussion

### Hygroscopicity and cloud activation potential

**Figure 1:**
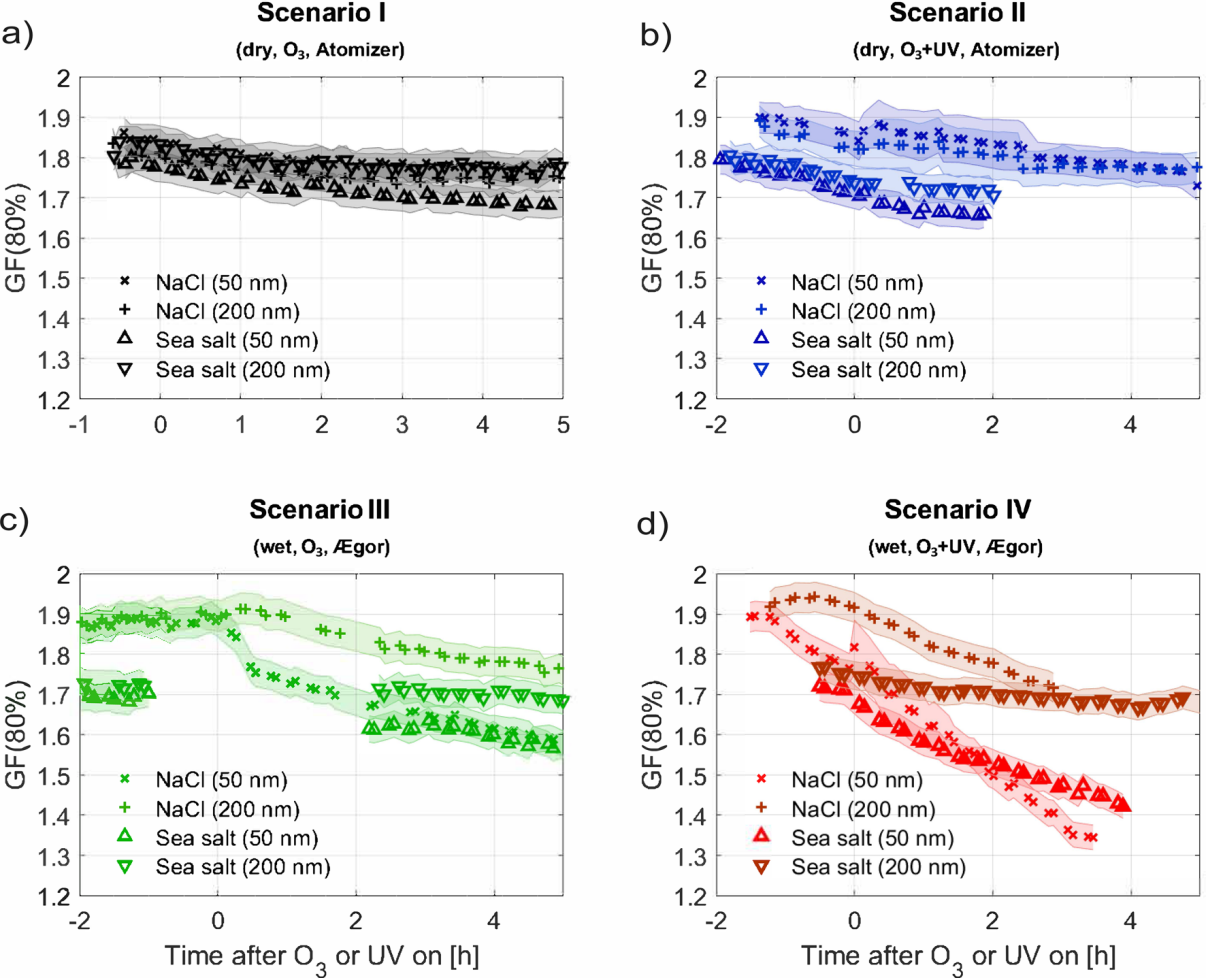
Effect of sea salt ageing on hygroscopicity. Growth factor (GF) measured at RH = 80% for NaCl and sea salt for dry particle sizes of D_dry_ = 50 nm and 200 nm. (**a**) Scenarios I (dry particles, dry chamber, $\hbox {O}_3$); (**b**) Scenario II (dry particles, dry chamber, $\hbox {O}_3$, UV); (**c**) Scenario III (humid conditions, $\hbox {O}_3$); (**d**) Scenario IV (humid conditions, $\hbox {O}_3$, UV). The 0-time-point marks the exposure start time to $\hbox {O}_3$ or OH. During t < 0, no oxidant was present in scenario I and III, while particles together with $\hbox {O}_3$ were present in the scenarios II and IV. The uncertainties represent the combined uncertainties of the GF(80%) measurement itself and the standard deviations from averaging the different experiments.

Sea spray aerosols were generated from aqueous solutions of artificial sea salt (termed *sea salt* from now on; salinity of 3.5%) or NaCl using a circular plunging jet^23^ or an atomiser and injected into a 5 m^3^ Teflon chamber^47^ at 291 K. Typical particle number concentrations were of the order of 5000 and 15000 #/cm^3^ at the beginning of the experiments using the plunging jet and atomiser, respectively. The water uptake by the salt particles was examined with a humidified tandem differential mobility analyser, a cloud condensation nucleus counter and a humidified nephelometer. Four different scenarios were investigated: I) particles in the dry chamber exposed to $\hbox {O}_3$, II) particles in the dry chamber exposed to $\hbox {O}_3$ and UV lights, III) liquid particles in the humid chamber exposed to $\hbox {O}_3$ and IV) liquid particles in the humid chamber exposed to $\hbox {O}_3$ and UV lights. In scenarios I) and II) particles will be either crystalline or amorphous, where NaCl was shown to be crystalline below 70% RH^48^ while sea salt particles could be amorphous even at very low RH^48^. Table 1 provides an overview of experimental conditions. Figure 1 shows hygroscopic growth factors at RH = 80% (GF(80%)) for NaCl and sea salt particles as a function of time during the performed experiments using Ægor, i.e. the plunging jet technique, and the atomiser. The data are presented as a mean over separate experiments carried out at the same conditions in the chamber (Exp. 1–19 in Table 1). The uncertainties represent the combined uncertainties of the GF(80%) measurement itself and the standard deviations from averaging the different experiments. Details for each single experiment, including the RH development in the chamber, particulate surface area, as well as humid results from atomiser experiments, are presented in the supplementary information (SI, Figs. S1, S2 and S3).

**Table 1:** Experimental conditions.

Exp. #	Date	Compound and generation technique	RH chamber [%]	T chamber [$^{\circ }$C]	Conditions	$\hbox {O}_3$ conc. [ppb]	Scenario	$\Delta$ GF [$\hbox {h}^{-1}$]
1	10.04.2018	NaCl—Jet	100	18	O3, UV	150	IV	– 0.16
2	12.04.2018	NaCl—Jet	100	18	O3, UV	150	IV	– 0.11
3	17.04.2018	NaCl—Jet	100	18	O3	150	III	
4	12.06.2018	NaCl—Atomizer	0	18	O3	150	I	
5	15.06.2018	NaCl—Atomizer	0	18	O3, UV	150	II	
6	18.06.2018	NaCl—Atomizer	70	18	O3, UV	150	IV	– 0.08
7	17.08.2018	NaCl—Atomizer	70	18	O3	150	III	
8	21.08.2018	NaCl—Atomizer	70	18	O3	150	III	
9	19.09.2018	NaCl—Atomizer	70	18	O3, UV	150	IV	– 0.10
10	09.07.2019	NaCl—Atomizer	70	18	O3, UV	150	IV	– 0.14
11	03.10.2018	Sea Salt—Jet	100	18	O3, UV	150	IV	– 0.06
12	07.10.2018	Sea Salt—Jet	100	18	O3, UV	150	IV	– 0.07
13	10.10.2018	Sea Salt—Jet	100	18	O3	150	III	
14	22.06.2018	Sea Salt—Atomizer	70	18	O3	150	III	
15	25.06.2018	Sea Salt—Atomizer	70	18	O3, UV	150	IV	– 0.07
16	27.06.2018	Sea Salt—Atomizer	70	18	O3, UV	300	IV	– 0.06
17	25.07.2019	Sea Salt—Atomizer	70	18	O3, UV	150	IV	– 0.06
18	12.10.2018	Sea Salt—Atomizer	0	18	O3, UV	150	II	
19	08.07.2019	Sea Salt—Atomizer	0	18	O3	150	I	

The temperature in the chamber was set to 18$^{\circ }$C during all experiments, but during experiments with UV lights on it increased to 20$^{\circ }$C.The GF(80%) decay rates ($\Delta$GF) for particles with D_dry_ = 50 nm per hour are indicated for experiments with ageing by exposure to $\hbox {O}_3$ and UV lights and are based on a linear regression of the change in GF(80%) during the first three hours of ageing.

During the first part of the experiments (i.e. t < 0) the GF(80%) for NaCl particles (dry diameters (D_dry_) of 50 and 200 nm) are in the range 1.78–1.90 in all experiments and scenarios and somewhat lower, in the range 1.68–1.80 for sea salt particles. The difference in GF between NaCl and sea salt is in agreement with the recent finding that the use of NaCl in experiments or in models overestimates the GF of sea salt^16^. Our findings coincide well with previously published GF values, recalculated for RH = 80% using $\kappa$-Köhler theory^49^, of 1.81–1.87 (using a nebulizer to generate the particles and calculated from Köhler theory) and 1.72–1.78 (using a plunging jet and a nebulizer), for NaCl and sea salt, respectively^16^.

For experiments performed at dry conditions the NaCl and sea salt particles are expected to be crystalline or amorphous in the smog chamber (scenarios I and II, Fig. 1a,b). A slight decrease in GF(80%) with time after exposure to $\hbox {O}_3$ and UV radiation is observed for both salts (max. 7% after 5 hours). This implies chemical and/or physical changes of the particles which at first seem hard to reconcile. They are however consistent with previous findings observing the formation of small amounts of $\hbox {Cl}_2$ when dry sea salt particles were exposed to $\hbox {O}_3$ and UV light, which were associated with chemical reactions taking place in a film of surface adsorbed water^42^. Such films can be present on dry NaCl and sea salt even after heating and pumping^50,51^. Sea salt furthermore contains salts that are hydrated also after drying, e.g. $\hbox {MgCl}_2\cdot 6\hbox {H}_2\hbox {O}$^17,18^, providing a liquid layer for surface chemistry^52^. Exposure of dry NaCl to $\hbox {O}_3$ has also been reported to lead to perchlorate formation, which may have an effect on hygroscopicity^53,54^.

For particles aged under humid conditions with $\hbox {O}_3$ (no UV light) some decrease in GF(80%) with time is observed (scenario III, Fig. 1c). There is some debate as to the detailed chemistry taking place when aqueous NaCl or sea salt is exposed to $\hbox {O}_3$ in the dark, the formation of chlorates was for example proposed^54–56^ which might influence hygroscopicity.

A remarkable result in Fig. 1 is the large decrease in GF(80%) when small (50 nm) liquid NaCl and sea salt particles are aged in the presence of $\hbox {O}_3$ and UV lights, which we ascribe to a strong effect of OH chemistry on hygroscopicity (scenario IV, Fig. 1d). After three hours of ageing, the GF(80%) reaches values of 1.38 and 1.46 for NaCl and sea salt, respectively. For the larger particles (200 nm) the GF(80%) after three hours of ageing stays relatively constant reaching values of 1.72 and 1.69 for NaCl and sea salt, respectively. Both particle sizes have been aged in the same way and thus the difference in hygroscopicity shows that the small particles, which have a higher surface to volume ratio, are significantly more aged indicating surface processes being responsible for this finding^52^.

**Figure 2:**
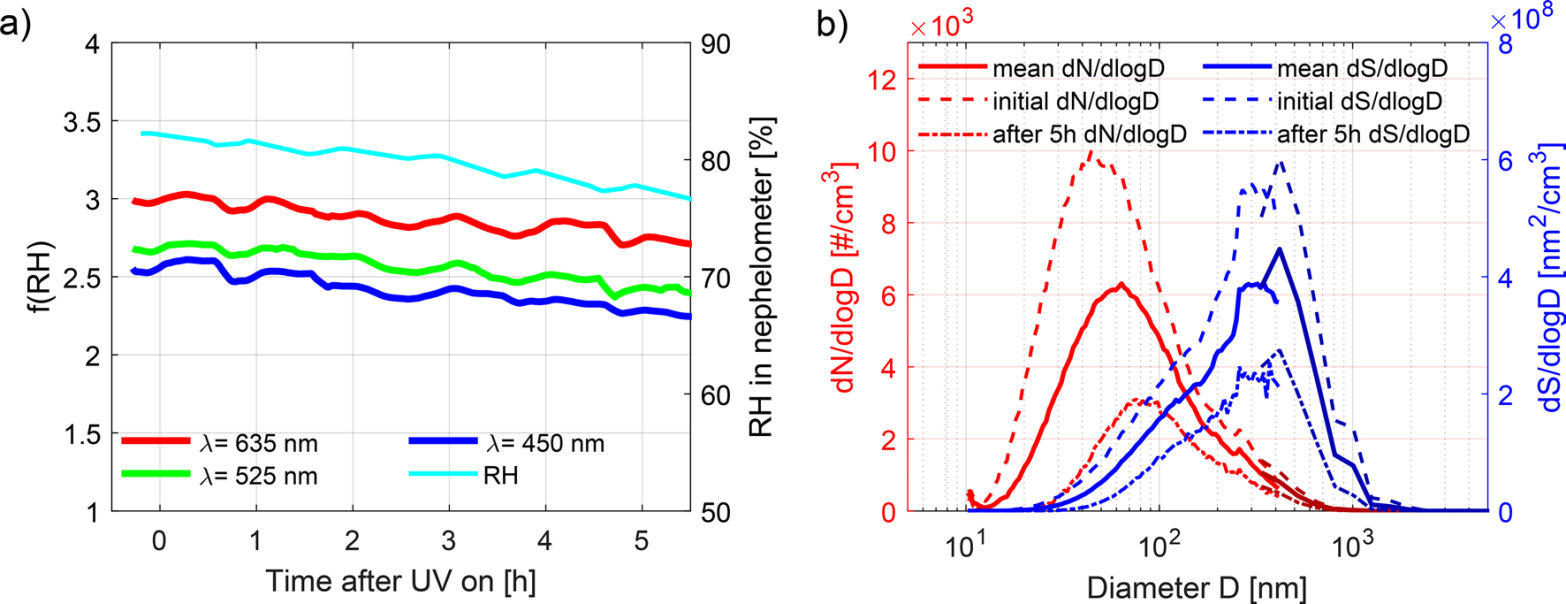
Hygroscopicity of larger sized sea salt particles (Exp. 15). (**a**) Scattering enhancement factor (f(RH)) measured at 3 different wavelengths ($\lambda$ = 450 nm, $\lambda$ = 525 nm, $\lambda$ = 635 nm) and RH in the nephelometer. (**b**) number (red lines; left y-axis) and surface distributions (blue lines; right y-axis) at the beginning, after 5 hours and mean distributions throughout the experiment as derived from combined scanning mobility particle sizer and optical particle spectrometer measurements.

The weak response of the large particles (200 nm) to chemical ageing was confirmed by measurements of the scattering enhancement factor (f(RH), defined as the ratio between wet and dry scattering coefficient) of the polydisperse sea salt distribution in the chamber (Exp. 15), suggesting no change in the particles’ optical properties with ageing. This optical analysis relies mainly on the particle surface area and is hence dominated by larger particles (mode diameter of surface distribution during experiment: $\sim$300 nm, Fig. 2b). The scattering enhancement factor f(RH) in Fig. 2a shows a small decrease over time (f(RH) from 3 to 2.5 at $\lambda$ = 635 nm) which can however, be accounted for by the change in RH from about 82% to 75% during the experiment. HTDMA and microscopy results indicate minor amounts of hydrate forming salts for particles of approximately 300 nm and thus a significant change in the particles’ dry index of refraction during ageing for this size seems unlikely. The size and surface area distributions (Fig. 2b) further illustrate that while the mode of the surface distributions remains relatively constant throughout the experiment, a substantial number and surface area of the particles are lost which according to model simulations can be ascribed to wall losses, including sedimentation, and coagulation (more details on the model are presented below). However, this will not affect the qualitative particle properties investigated in this study.

**Figure 3:**
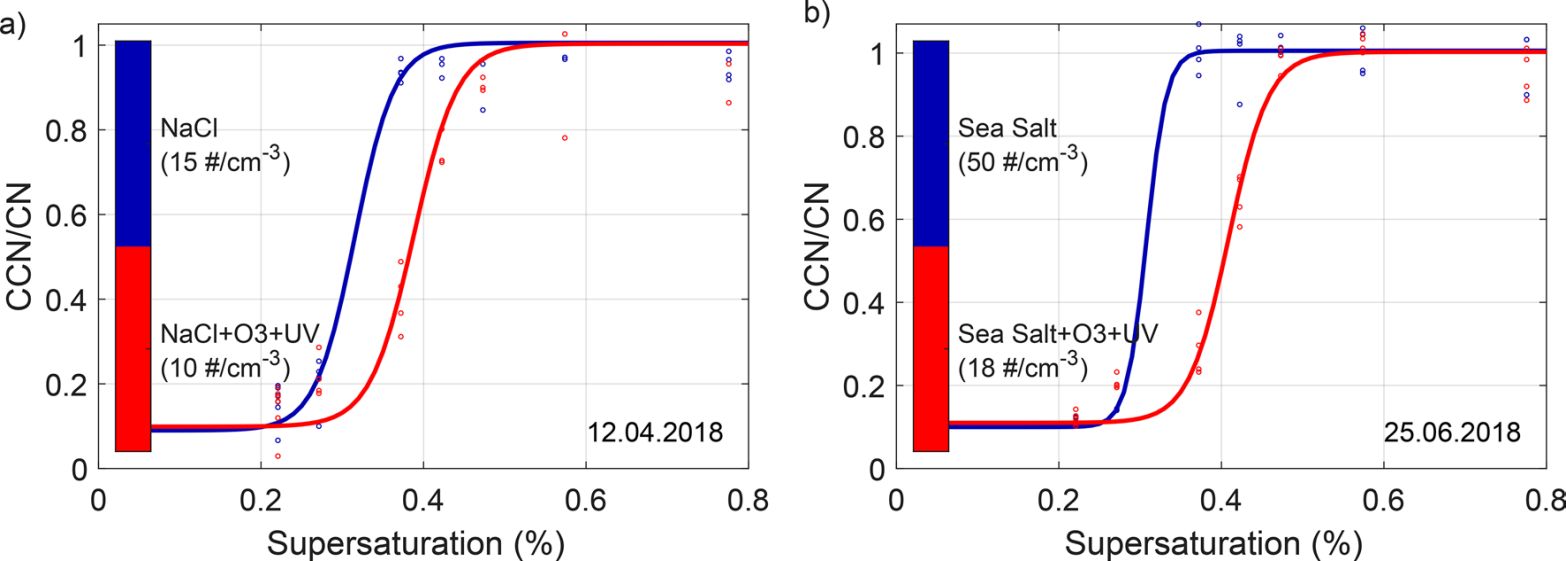
Effect of ageing on CCN activation potential. Measurement of the critical supersaturation for monodisperse particles of D_dry_ = 50 nm. Results for NaCl are illustrated in (**a**) and for sea salt in (**b**). The blue lines show CCN activation at the initial state of the experiments (fresh NaCl and SSA), while the red lines illustrate CCN activation after 3 hours of ageing in the presence of $\hbox {O}_3$ and UV lights (aged NaCl and SSA).

Simultaneously, the critical supersaturation (SS_crit_) for dry, monodisperse 50 nm particles was measured (Exp. 2, 14). This diameter falls in the key size range relevant for supersaturations typically experienced in cloud systems in oceanic regions^57^. The results in Fig. 3 show that fresh NaCl particles (a, blue line) are activated slightly earlier (SS_crit_ = 0.32) than fresh sea salt (b, blue line; SS_crit_ = 0.33). The results for fresh NaCl and sea salt are consistent with previously published data of SS_crit,NaCl_ = 0.32–0.33^58,59^ and SS_crit,sea salt_ = 0.34–0.36^59,60^. After three hours of ageing by $\hbox {O}_3$ and UV lights, the SS_crit_ needed to activate the particles is increased for both types of salts reaching values of SS_crit_ = 0.39 (a, red line) and SS_crit_ = 0.41 (b, red line), for NaCl and sea salt, respectively. Hence, the cloud activation potential of such SSA is evidently reduced by the simulated ageing. These findings are in accordance with the hygroscopicity results presented above.

Although all experiments were performed with particles generated from inorganic salts, we cannot fully exclude a small contamination by organic compounds. We find that it is highly unlikely that organic particle mass can be responsible for the observed changes in hygroscopicity and cloud activation for the following reasons: First, the water uptake potential of dry salt particles was measured with three independent methods yielding values that are well comparable with literature data, indicating negligible contamination. Second, calculations to assess the amount of organics needed to reach the values found in scenario IV for 50 nm particles show that the organic fraction has to be at least 70% to account for a lowering of the growth factor from 1.9 to 1.4 (see Fig. S4), which is very unlikely. Third, a contamination by organic compounds in the initial stages of the experiment by e.g. being present in the used salt solution would yield lower GF values right from the beginning. Organic contamination in later stages would be oxidised through ageing typically leading to an increase in hygroscopicity but it would still be much less hygroscopic compared to the salt particles. Forth, simulations with the Aerosol Dynamics gas- and particle-phase chemistry model for laboratory CHAMber studies (ADCHAM)^61,62^ revealed that the increase in particle diameter over time, as visible from Fig. 2b, can be fully explained by coagulation and particle wall losses and is not a sign of organic mass condensation (see Figs. S5 and S6 in SI). Specifications on the model settings are described in the SI.

Even though all experiments were carried out at low nitrogen oxide (NO_x_) levels, a small amount of NO_x_ could lead to the formation of sodium nitrate (NaNO_3_) inside the particles during high humidity experiments^63^. The GF(80%) for 50 nm NaNO_3_ particles is 1.58^64^, which is much lower compared to that of NaCl and SSA and could lead to a decrease in the overall GF. As it is, however, higher compared to the measured GF(80%,50 nm) of approximately 1.4 after four hours of ageing, even a particle containing only NaNO_3_ cannot explain the presented results of aged NaCl. Results of the elemental composition also do not suggest the presence of NaNO_3_ (see discussion below).

### Elemental composition

**Figure 4:**
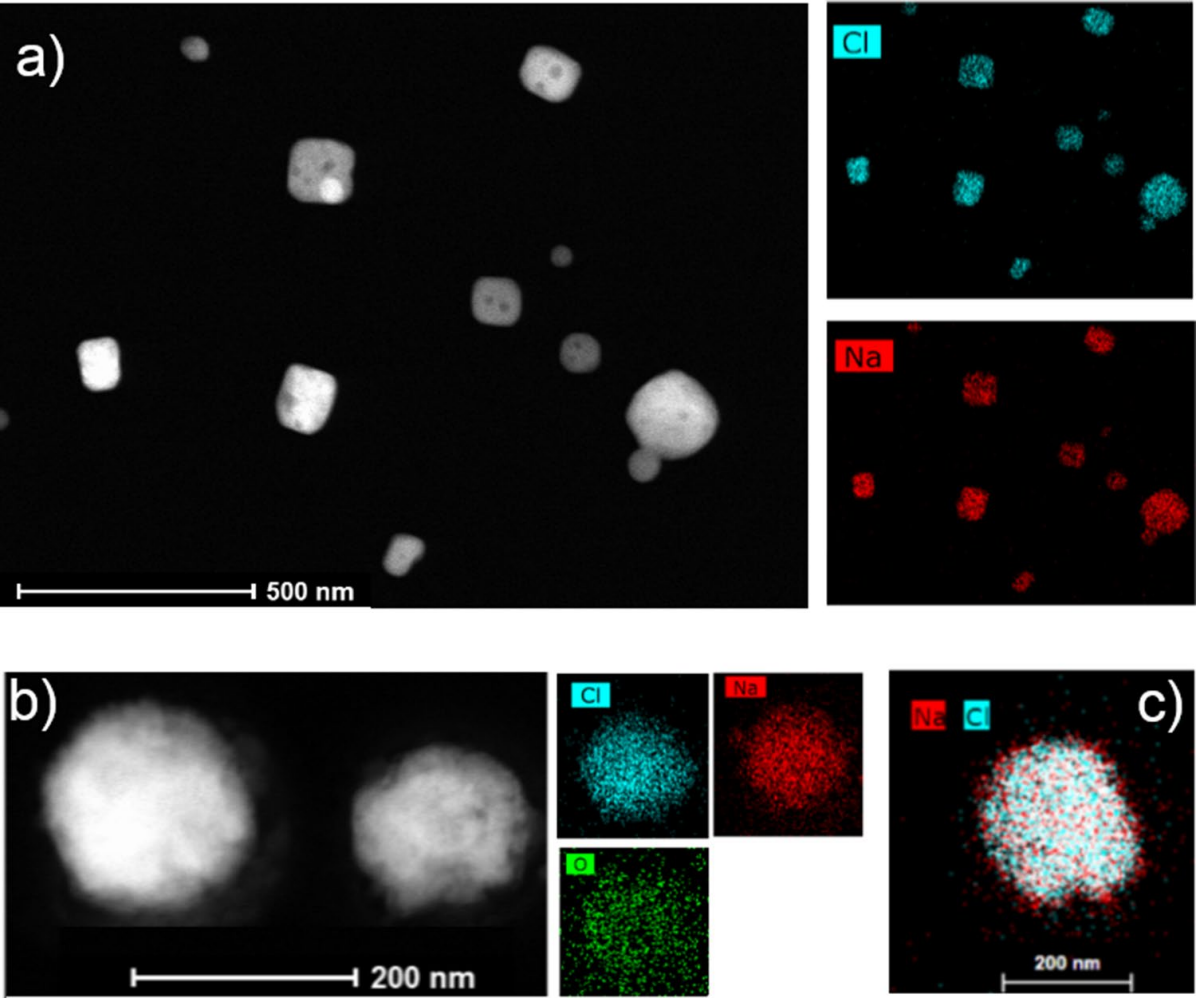
Effect of ageing on particle elemental composition. Microscopy images (STEM-EDX) of fresh (**a**) and aged (**b**) NaCl particles. Additionally, the elements found in the fresh and aged particles are illustrated. c) overlap of Na and Cl signals in the aged samples.

Figure 4a shows microscopy images of fresh NaCl particles appearing relatively cubic with contributions from only Na and Cl (Exp. 2). The sample shows a distribution of particle sizes between approximately D_dry_ = 50 and 300 nm, all containing about 50% Na and 50% Cl (atomic %). After 3 hours of ageing (Scenario IV), the particles change shape from close to cubic at the beginning of the experiment to more spherical after ageing (Fig. 4b; Exp. 1). Such a change in shape can affect the hygroscopicity results that are based on the comparison of dry and wet volumetric diameters. The diameters are measured with a differential mobility analyser (DMA). For spherical particles the mobility and volumetric diameters coincide while non-spherical particles need to be corrected with a dynamic shape factor to account for differences in shape. According to Zieger et al.^16^ fresh salt particles generated in the laboratory that appear cubic-like need a dynamic shape correction factor of approximately 1.10 to recalculate volumetric particle diameters from the measured mobility diameters. By applying this correction, the GF values increase by approximately 10%. Therefore, the GF(80%) illustrated in Fig. 1 at t < 0 (fresh particles) present lower estimates. The GF(80%) of the aged particles (t > 0) do not need any correction as aged particles have a more spherical shape.

Additionally, the aged particles exhibit a decrease in Cl content and a concurrent increase in O content. Similar findings were previously published, displaying a replacement of Cl by O mainly for particles below 1 $\mu$m (^46^, measurements were carried out above D_dry_ = 150 nm). Laskin et al.^46^ attributed the Cl decrease to emissions of molecular chlorine to the gas-phase, while they proposed the formation of NaOH in the particulate phase due to reactions with OH radicals. Another possibility for the appearance of O is the formation of chlorates^55,56^. By overlapping the signals for Na and Cl a depletion of Cl can be seen primarily at the surface of the particles (Fig. 4c; Exp. 2).

The elemental composition can also be used to infer a possible contamination by e.g. organic matter or NaNO_3_. Results for fresh NaCl particles (Fig. 4a) do not suggest the presence of either of them at any of the selected sizes as no oxygen was measured. The increased oxygen signal for the aged particles (Fig. 4b) could potentially also originate from organic matter or NaNO_3_. The concurrent Cl depletion does not support the presence of organics but rather that of NaOH or chlorates as explained above. A detailed microscopy study on NaCl/NaNO_3_ mixtures by Hoffman et al.^65^ revealed that NaNO_3_ is typically found as a shell around the particles producing an oxygen signal concentrated around the particles rather than inside. Although some of NaNO_3_ could have evaporated during our microscopy measurements, results of aged NaCl in Fig. 4 do not suggest a core–shell structure but an evenly distributed oxygen signal. Finally, Fig. 4b,c shows that aged NaCl particles are still predominately composed of Na and Cl, thus suggestion a very limited potential presence of NaNO_3_.

**Figure 5:**
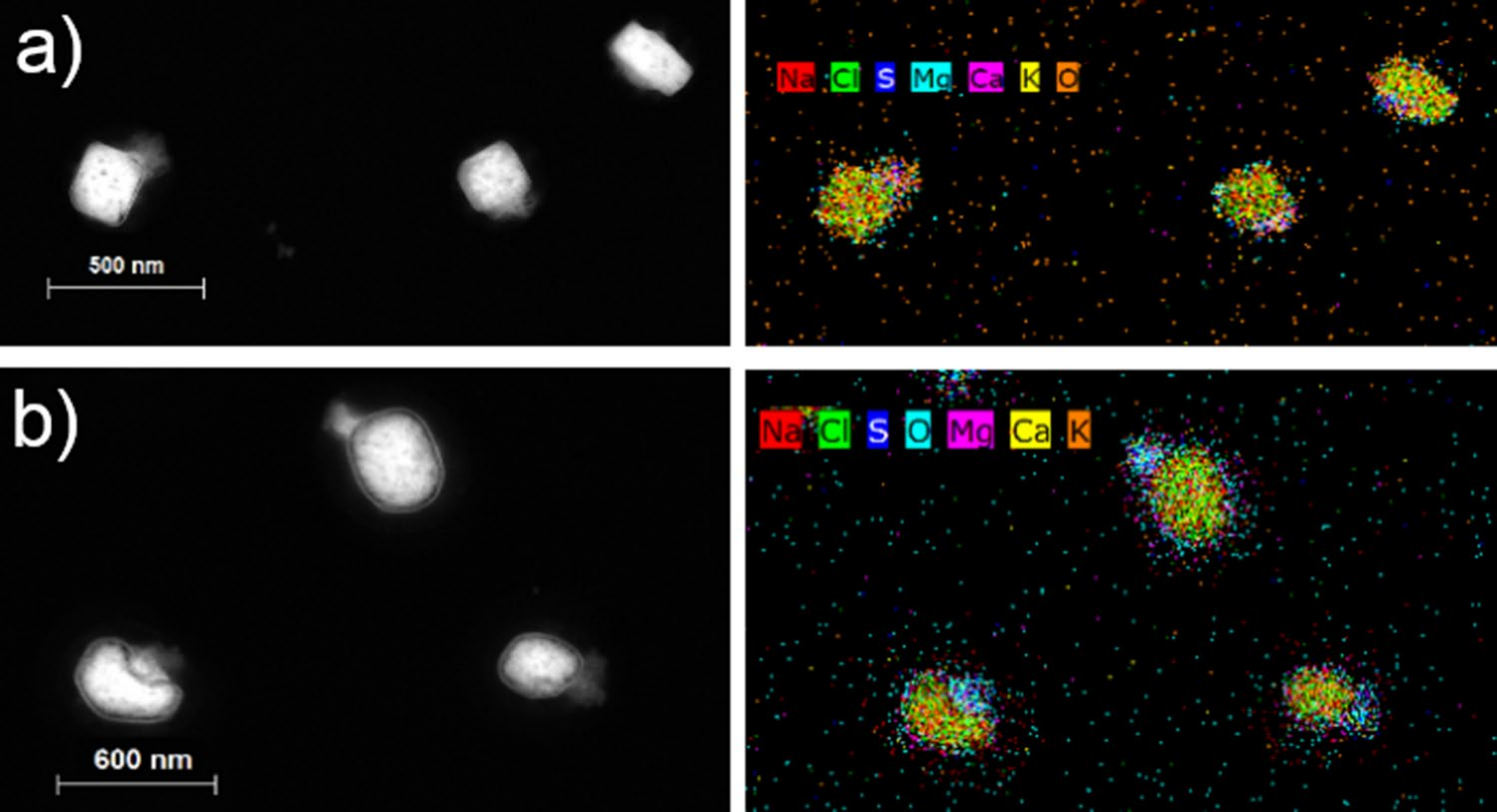
Effect of ageing on shape and elemental composition. Microscopy images (STEM-EDX) of fresh (**a**) and aged (**b**) sea salt particles, illustrating the shape and elemental compositions found in these particles.

The analysis of sea salt reveals that while the fresh particles appear relatively cubic (Fig. 5a; Exp. 11), the aged ones have more round edges (Fig. 5b; Exp. 11). Furthermore, for both the fresh and aged particles, the core mainly consisted of Na and Cl, while S and O are visible in little pockets at the surface and Mg is present around the core of the particles, which coincides with measurements of real sea spray samples^7,37^. The presence of oxygen in fresh sea salt particles is expected considering the composition of the salt chosen to generate such aerosols (more information can be found under “Materials and methods”). The concentrations of Ca and K were not sufficient for an accurate analysis.

### Hydrates

**Figure 6:**
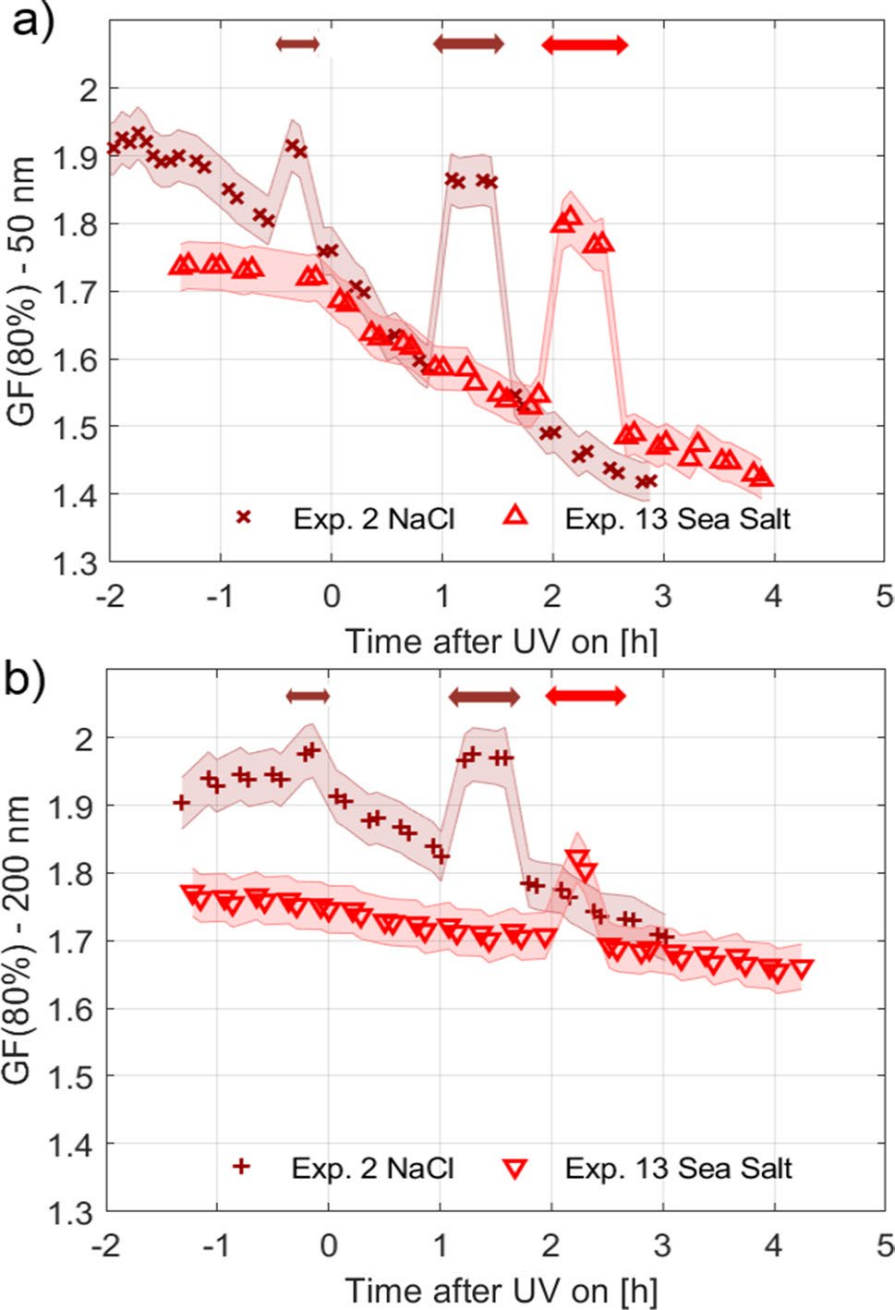
Effect of heating the aerosol particles. Growth factors at RH = 80% measured for NaCl and sea salt for the two different sizes of D_dry_ = 50 nm (**a**) and 200 nm (**b**) are shown. The influence of heating the particles to 300$^{\circ }$C, additionally to drying them, is illustrated. The arrows indicate the time period when the heating was switched on.

Dried NaCl is present in its anhydrous form at 291 K^66^. During exposure to OH the chemical composition of NaCl in aqueous solution changes, leading to a depletion in Cl and replacement by O^46^. Therefore, after ageing, the aqueous particles may contain newly formed compounds like NaOH and $\hbox {NaClO}_x$ (chlorates). These compounds are known to exist in hydrated forms upon drying^67^, implying that even drying to very low RH cannot evaporate all water molecules, keeping a fraction bound to the salt. While NaOH is known to be present as mono- up to heptahydrate^68^, NaClO is known to mainly exist as pentahydrate^69^ and $\hbox {NaClO}_4$ as monohydrate^69^. The GF(80%) for 50 nm NaCl particles was shown to continuously decrease nearly linearly after ageing, indicating a change in the chemical composition within the droplets (Fig. 1). The presence of hydrated salts can explain this phenomenon, as when exposed to high RH a smaller amount of water can be taken up due to the fraction of water molecules already trapped within the crystal. To test this hydrate hypothesis, we dried (RH < 10%) and subsequently heated the NaCl particles with a thermodenuder, set to a temperature of 300$^{\circ }$C, before exposing them to RH = 80%. The results in Fig. 6a (Exp. 2 and 11) illustrate that this procedure leads to an immediate change in GF(80%) for D_dry_ = 50 nm. Each time the heating is turned on, aged and heated NaCl (dark red) increases its GF(80%) to values comparable to those for the fresh NaCl particles. Microscopy images of NaCl after the thermodenuder treatment indicate mainly contributions of Na and Cl, while O could not be detected (Fig. S7).

Sea salt is a complex inorganic mixture containing compounds like $\hbox {MgCl}_2$ and $\hbox {CaCl}_2$, which are known to exist in hydrated forms under various conditions^67^. This was recently suggested to influence the interpretation of thermodenuder data^17^ and to be the reason for the slightly lower hygroscopicity of fresh sea salt particles compared to pure NaCl particles^16^. This small difference is also visible in this study (Fig. 6). When sea salt is dried (RH < 10%) and additionally heated to 300$^{\circ }$C, the GF(80%) increases rapidly to values slightly higher than those for fresh sea salt particles. This implies that at 300$^{\circ }$C, water bound as hydrates in the compounds formed during ageing, but also hydrates present even in the freshly generated sea salt particles are evaporated.

The GF(80%) of particles with D_dry_ = 200 nm (Fig. 6b) is also affected by the heating process, reaching values comparable to those at the beginning of the experiment in the case of NaCl and slightly higher values for sea salt. In the marine atmosphere, SSA may exist as aqueous phase solution droplets and to investigate its hygroscopicity, for example using an HTDMA, the particles have to be dried most certainly leading to formation of several hydrated salts^18^. The decreasing hygroscopic growth factors with time for the same dry particle size observed in our study are ascribed to ageing reactions in the aqueous droplets leading to formation of hydrate forming salts and as a result hydrates in the dried particles. There is no real reason to assume that formation of hydrate forming salts would not take place also in the marine boundary layer with time. Since SSA may undergo several RH cycles during their atmospheric transit^70^, hydrate forming salts are expected to have an effect on the aerosols’ physical and chemical properties and ultimately their role in climate.

## Conclusion and implications

The unique setup combining an environmental chamber with a sea spray simulation tank directly targets the evolution of SSA affected by different ageing mechanisms. We follow the common practice of laboratory and field studies and report hygroscopic growth and cloud forming potential based on dried size-selected particles. This study reports key findings on inorganic SSA ageing by exposure to $\hbox {O}_3$ and UV lights, yielding OH radicals, that are crucial in daytime chemical reactions. Our results suggest that ageing processes in aqueous sea spray solution droplets induce surface reactions, which significantly alter the hygroscopic growth and cloud activation potential of the dried particles. In particular we observed an effect on the smaller particles (D_dry_ = 50 nm), while the effect on larger ones (D_dry_ = 200 nm) is minimal. The lowering of the growth factor and CCN activation potential after ageing is explained by a change in the amount of water retained under dry conditions as a result of hydrate formation. To relate these results to ageing of SSA under atmospheric conditions four key parameters have to be compared: the prevailing RH, $\hbox {O}_3$, OH and aerosol number concentrations. During our simulated ageing, the RH was kept at values that are representative for the marine boundary layer (> 60%), while the $\hbox {O}_3$ and aerosol number concentrations were somewhat higher: 150 ppb $\hbox {O}_3$ and 2000–20000 #/$\hbox {cm}^3$ in our experiments vs. typically 10–60 ppb $\hbox {O}_3$^71^ and < 1000 #/$\hbox {cm}^3$^72^ in the clean marine boundary layer. Although the particle number concentrations and hence particle surface in the chamber was substantially higher compared to the real atmosphere, the water uptake properties of sea salt and NaCl at different concentrations are comparable, suggesting that this did not considerably affect the herein presented results. Based on an experiment monitoring the decay of 1-butanol at elevated RH ($\sim$60%), $\sim$150 ppb $\hbox {O}_3$ and using UV lamps to initiate the photolysis of $\hbox {O}_3$ we estimate the OH concentration to be in the range of $\sim 10^6$ molecules/$\hbox {cm}^3$ or lower (for more information see "Materials and methods") and hence values comparable to those found in the troposphere^1^. This agreement suggests that the measured GF(80%) decay rates are representative of changes occurring over the oceans due to ageing processes and do not need to be scaled to the degree the elevated $\hbox {O}_3$ levels would suggest, to represent in-situ conditions. The GF(80%) decay rates for NaCl and sea salt are presented in Table 1 (see also Fig. S8), as calculated from the linear decrease of each experiment during the first three hours of ageing. The results for sea salt are comparable between the repetitions and the two generation techniques amounting to $-0.06\pm0.01 \hbox { h}^{-1}$ on average, while a more pronounced and more variable decrease is found for NaCl reaching $-0.12 \pm 0.03$ $\hbox {h}^{-1}$ on average. General Circulation Models (GCM) assuming that sea salt aerosols behave like NaCl will thus overestimate their GF as previously suggested^16^, but our data suggests in addition that the assumption of similarity to NaCl will be more problematic with time if the models do not also include the ageing processes of sea salt: $\hbox {Cl}_2$ losses, oxidation and hydration.

The significant change in hygroscopicity, demonstrated in this work, and the formation of hydrate forming salts during atmospheric ageing has not been previously reported but could be of major importance for interpretation of the hygroscopic and cloud activation properties of marine SSA measured over the oceans, since such measurements are typically performed with dried particles as the baseline. The observations in the marine boundary layer state a prevalent MHP GF-group described as GF(90%,100nm) > 1.33, while the SSP group with GF(90%,100nm) > 1.85 occurred only at high wind speeds^32,33^. By using $\kappa$-Köhler theory^49^ to recalculate the GFs measured in this laboratory study for sea salt at RH = 90%, values of GF_fresh_(90%,50nm) = 2.18 ($\kappa$ = 1.20) and GF_aged_(90%,50nm) = 1.73 ($\kappa$ = 0.59) (after 3 hours of ageing) are found. These results are also in good agreement with $\kappa$-values calculated from SS_crit_ yielding $\kappa$_fresh_ = 0.99 and $\kappa$_aged_ = 0.65 for sea salt particles with D_dry_ = 50 nm.

When applying the above mentioned GF-categories, the fresh particles would hence be classified as SSP and the aged ones as MHP. Therefore, our results suggest that the dominant MHP-group over the oceans could be explained by the presence of aged inorganic SSA leading to an increased amount of hydrate forming salts within the particles and that these observations are compatible with sea spray being a major aerosol source in marine regions. It has to be noted however, that real SSA is not solely composed of inorganics (like sea salt used in this study) and thus other suggested alternatives comprising the presence of sulphate and organic matter might also play a role. On the other hand the argument that GFs in the MHP-range disprove the presence of sea salt in favour of biogenic sulphates and organics is invalid. Indeed, these compounds could contribute to a lower GF, but the amount of biogenic sulphate and organics change with the biological production and thus with season and location, while sea salt is always present as long as there is some wind^33^, pointing to aged sea salt being a key explanation for the lower GFs.

We also show that slightly larger particles (D_dry_ = 200 nm) are not equally affected by the same amount of ageing. The analysis of the chemical composition using microscopy techniques accentuates processes occurring mainly at the surface of the particles, which are more decisive for smaller sizes and could explain the differences found for the two dry particle sizes after ageing, as particles with D_dry_ = 50 nm are four times as easily changed in bulk composition due to surface processes than 200 nm particles. The new oxygen containing compounds formed in aged particles initially containing only NaCl, are most likely NaOH and Na-chlorates. Both are known to form hydrates at low RH, which strongly influence the hygroscopic properties of particles. By additionally heating the particles to 300$^{\circ }$C, the initial GF(80%) was restored, suggesting that this temperature was sufficient to decompose the hydrated compounds leaving a particle predominately containing NaCl behind. Hydrates hinder the water uptake as they already contain water molecules in their crystalline structure reducing the amount of water that can be taken up at elevated RH. In the case of the more complex (inorganic) sea salt mixture some of the compounds, especially $\hbox {MgCl}_2$ and $\hbox {CaCl}_2$, are known to exist in their hydrated form in the atmosphere. The ageing effects on the complex sea salt mixture are comparable to those of NaCl exhibiting a substantial decrease in hygroscopicity and CCN activity and can, again, be reversed by drying and additionally heating the particles before exposing them to high RH. Thus, there is a need to further investigate atmospheric ageing of sea spray aerosol and examine uncertainties arising from likely overestimations of the particles’ hygroscopicity and ability to form CCN in climate models and adapt the parametrizations for sea salt aerosols accordingly.

## Materials and methods

### Experimental procedure

All experiments were performed at the AURA smog chamber facility^47^. Before each experiment, the Teflon chamber was filled to approximately 5 m^3^ with purified dry air provided by passing compressed air through first an active carbon and HEPA filter and second a zero-air generator (Aadeco model 737-14) to clean for volatile organic compounds, particles and residual gases. The chamber was kept at atmospheric pressure and a temperature of 293 K. Two techniques were used to generate SSA: a temperature controlled sea spray simulation tank Ægor^23^ and an atomiser (TSI model 3076). Ægor was operated with a plunging jet (nozzle diameter 4 mm) using a jet water flow rate of 5 l/min. Sweep air was pushed through the head-space with a flow rate of 10 l/min and transferred to the AURA smog chamber via stainless steel tubing. Transfer continued until the RH inside the Teflon chamber reached RH > 90%. This is above the deliquescence point of the chosen salts and thus it was ensured that the SSA existed in the chamber as aqueous solution droplets. SSA generation with the atomiser was very efficient and hence an external custom-made humidifier was employed to pre-condition the chamber to RH > 70%. Then SSA droplets from the atomiser were injected into the chamber for one minute without drying. In this set-up liquid SSA was ensured by keeping RH above the efflorescence point of the salts (47% for NaCl^16,28,73^, 50% for sea salt^16^). For the dry experiments the RH in the chamber was kept close to 0% and SSA was injected using the atomiser. The salts chosen for the experiments were pure NaCl (Sigma Aldrich; purity > 99.5%) and artificial sea salt (Sigma Aldrich S9883; mass fraction: 55% $\hbox {Cl}^{-}$, 31% $\hbox {Na}^{+}$, 8% $\hbox {SO}_{4}^{2-}$, 4% $\hbox {Mg}^{2+}$, 1% $\hbox {K}^{+}$, 1% $\hbox {Ca}^{2+}$, < 1% other; termed sea salt throughout the manuscript). In all experiments the salts were dissolved in MilliQ water (EMD Millipore, 18.2 M$\Omega \cdot$cm at 25$^\circ$C resistivity, 2 ppb TOC) achieving a salinity of 3.5% (by mass).

The experiments proceeded through three stages: In the first stage the smog chamber contained the SSA in either a humid or dry atmosphere. In the second stage approximately 150 ppb of $\hbox {O}_3$ (ozone generator Model 610, Jetlight Company, Inc.) were injected and dark chemistry was allowed to occur. In the third stage, daytime chemistry was simulated by turning on UV lamps photolysing $\hbox {O}_3$ (wavelengths from 300–400 nm) thereby producing OH radicals. $\hbox {O}_3$ and $\hbox {NO}_x$ concentrations were continuously monitored using a UV absorption ozone analyser (O342 Module, Environment S.A) and a chemiluminescent monitor (AC32M, Environnement S.A), respectively. The $\hbox {NO}_x$ levels were always < 5 ppb. Particle size distributions (D_dry_ = 10–450 nm) were measured using a scanning mobility particle sizer (SMPS) comprising a Kr-85 neutraliser (TSI 3077A) and an electrostatic classifier (TSI 3082) coupled with a nano water-based condensation particle counter (CPC; TSI 3788), while sizes between D = 0.35–10 $\mu$m were measured with an optical particle spectrometer (OPS; TSI 3330). A silica gel diffusion dryer was placed in front of each instrument to record dry size distributions. To reconcile SMPS and OPS measurements, the optical diameters determined from the OPS were recalculated for an index of refraction of m = 1.54 representative for NaCl^74^ and assumed to be the same for the artificial sea salt mixture^75^.

### Hygroscopicity and cloud activation instruments

The hygroscopic growth of particles with D_dry_ = 50 and 200 nm was measured with a humidified tandem differential mobility analyser (HTDMA; Brechtel, Model 3002^76^). The RH for dry particle measurement was constantly monitored and always kept < 10%, while humid experiments were all carried out at 80% RH. The accuracy of the HTDMA was verified with measurements using ammonium sulphate (Sigma Aldrich, purity > 99.95%). The combination of instrumental uncertainty (estimated to be 2% sizing accuracy and 1% RH uncertainty) and deviation from theoretical values calculated with Köhler theory (max. 2% for the chosen size range) lead to an overall measurement uncertainty of less than 3% in the GF(80%) values. A reliable correction for the shape of the particles was not possible in this analysis as microscopy results revealed an evolution of the particles’ shape with time. Thus, a different correction would be necessary for fresh (more cubic) and aged (more spherical) particles. It should be pointed out that we did not measure the amount of water in the aqueous solution droplets during ageing. Additionally, a thermodenuder (TD; Brechtel, Model 3105) was utilised in combination with the HTDMA. In this set-up the TD was set to a temperature of 300$^{\circ }$C and placed downstream of the HTDMA drier but upstream of the first DMA. In this way the hygroscopic behaviour of not only dried but also thermally treated particles was analysed.

Hygroscopicity was additionally probed with a commercially available humidified nephelometer system employing a three wavelength nephelometer (Ecotech Pty Ltd., Aurora 4000) together with an especially designed humidification system (aerosol conditioning system (ACS1000) by Ecotech Pty Ltd.). The original set-up was altered to contain solely one nephelometer and RH in the humidifier was alternating between 30% (dry) and 80% (wet) every 20 minutes to measure once dry and once humidified scattering coefficients. The scattering enhancement factor f(RH) can be calculated from the scattering coefficient $\sigma _s$ at dry and elevated RH: f(RH) = $\sigma _{s,wet}$/$\sigma _{s,dry}$. Measurements using the nephelometer were carried out using the polydisperse size distribution directly obtained from the smog chamber. The nephelometer was calibrated using particle-free air and $\hbox {CO}_2$ as a span gas and the system was additionally calibrated with ammonium sulphate (Sigma Aldrich, purity > 99.95%). The observed deliquescence RH coincided with theoretical values within 5%.

The CCN activity of the particles was monitored with a CCN counter (CCN 100, Droplet Measurement Technologies). Measurements were performed using a constant monodisperse diameter of D_dry_ = 50 nm, selected by a DMA (TSI, model 3081) keeping at sheath to aerosol flow of 1:10. After the DMA the aerosol flow was split between the CCN counter (0.5 l/min) and a CPC (0.3 l/min TSI, Model 3776). The supersaturation of the CCN counter was calibrated using dried ammonium sulphate particles (Sigma Aldrich, purity > 99.9999%) generated by an atomiser according to^77^, with theoretical values calculated using the E-AIM model (Extended-Aerosol Inorganics Model, http://www.aim.env.uea.ac.uk/aim/aim.php). The calibration curve obtained with ammonium sulphate was further used to infer the uncertainty of SS_crit_. For this purpose the method described in Harris et al.^78^ was applied, which is based on the uncertainty of the fit values, i.e. the slope and intercept. Overall, a mean uncertainty of ±0.02% was determined for the measured SS_crit_ values. In the data analysis the first 2 minutes at each supersaturation were omitted to ensure that temperatures in the column had stabilised. For the lowest supersaturation setting, the initial 5 minutes were omitted. The data was averaged on a per minute basis, the activation ratio calculated and normalised to unity to account for different particle losses and counting efficiencies of the CPC and CCN counter. The SS_crit_ was found by fitting a sigmoidal curve in MATLAB, where 50% had activated to CCN. We accounted for multiple charged particles by fitting from the level of the lower plateau in the spectrum following^77^.

### Microscopy

Samples for microscopy analysis were collected on pure carbon coated Ni-pure grids (CF400-NI-UL, EMS) with a Micro INertial Impactor (MINI;^79^) using one sampling stage with an aerodynamic cut-off diameter of either 50 or 60 nm. A flow of 0.5 l/min was utilised and the sampling time was approximately four minutes. Two samples were taken twice per experiment: two samples before any oxidant was added (fresh aerosol) and two after approximately 3 hours of exposure to OH radicals (aged aerosol). Scanning Transmission Electron Microscopy (STEM; FEI TALOS F200A, Thermo Fischer Scientific) was performed in combination with Energy-Dispersive X-Ray (EDX) detection to retrieve the elemental composition of the SSA and their shape. Carbon was not measured as it was already present on the used TEM grid. The STEM images were obtained using a high angle annular dark field detector (HAADF) and energy dispersive X-ray spectroscopy elemental maps were obtained using a Super-X EDS detector. The measurement of the elemental composition of 50 nm particles was not possible as such particles were not stable enough for this analysis and evaporated under the electron beam.

### Estimation of OH radical concentration during the experiments

We performed an additional experiment (3.3.2019) monitoring the decay of 1-butanol at elevated RH ($\sim$60%), $\sim$150 ppb $\hbox {O}_3$ and using the UV lamps in the AURA chamber to initiate the photolysis of $\hbox {O}_3$ leading to the formation of OH. Based on the reaction rate constant for 1-butanol and OH, known to be $8.47\pm 0.34 \times 10^{-12}$ $\hbox {cm}^3$ $\hbox {molecule}^{-1}$ $\hbox {s}^{-1}$ at 298 K^80^, we estimate OH concentrations in the range of $\sim 10^6$ molecules/$\hbox {cm}^3$ and hence values comparable to those found in the troposphere^1^. Although the chamber was carefully cleaned after each experiment, a possible influence by Cl radicals generated from leftover salt particles on the walls on the 1-butanol decrease rate cannot be fully excluded which makes the estimate an upper limit. The UV intensity might have varied between experiments due to failing of some of the lamps in the AURA chamber and this might influence the concentration of OH radicals available for reactions. It is, however, difficult to know the UV intensity in each experiment, as they were carried out over several months (April until October 2018). To ensure that this did not have a significant effect on the results, two ageing experiments were repeated with a complete set of new UV lamps (Exp. 9 and 17 in Table 1. An experiment without lamps was also repeated, Exp. 19). The data presented in Figs. S2 and S3, suggest a negligible influence as the results fall in the same range as those from previous experiments. This is also clearly visible in Fig. S8 where the GF decrease rates for particles of D_dry_ = 50 nm (also presented in Table 1) are displayed and compare well to those recorded in the other experiments.

## Supplementary information

Supplementary Information.

## Data Availability

The data obtained in this study and the codes for the analysis, written in Matlab, are available on request from the first author B.R. The ADCHAM model source code, which was used to analyse the impact of aerosol dynamics, wall losses and dilution effects, can be obtained upon request from P.R.
