# Supplementary material for: The impact of atmospheric oxidation on hygroscopicity and cloud droplet activation of inorganic sea spray aerosol
Source: Sci Rep. 2021 May 11;11:10008. doi: 10.1038/s41598-021-89346-6 (PMC8113565; doi:10.1038/s41598-021-89346-6)
Supplement: Supplementary file 1 — Supplementary Information. [file 41598_2021_89346_MOESM1_ESM.pdf]

Supporting Information:

The impact of atmospheric oxidation on hygroscopicity and cloud droplet activation of inorganic sea spray aerosol

Bernadette Rosati[1,*], Sigurd Christiansen[1], Anders Dinesen[1], Pontus Roldin[2], Andreas Massling[3], E. Douglas Nilsson[4], and Merete Bilde[1,*]

[1]Department of Chemistry, Aarhus University, DK-8000 Aarhus C, Denmark

[2]Division of Nuclear Physics, Lund University, SE-22100 Lund, Sweden

[3]Department of Environmental Science, University of Aarhus, DK-4000 Roskilde, Denmark

[4]Department of Environmental Science, Stockholm University, SE-11418 Stockholm, Sweden

[*]Corresponding Authors: bernadette.rosati@chem.au.dk, bilde@chem.au.dk

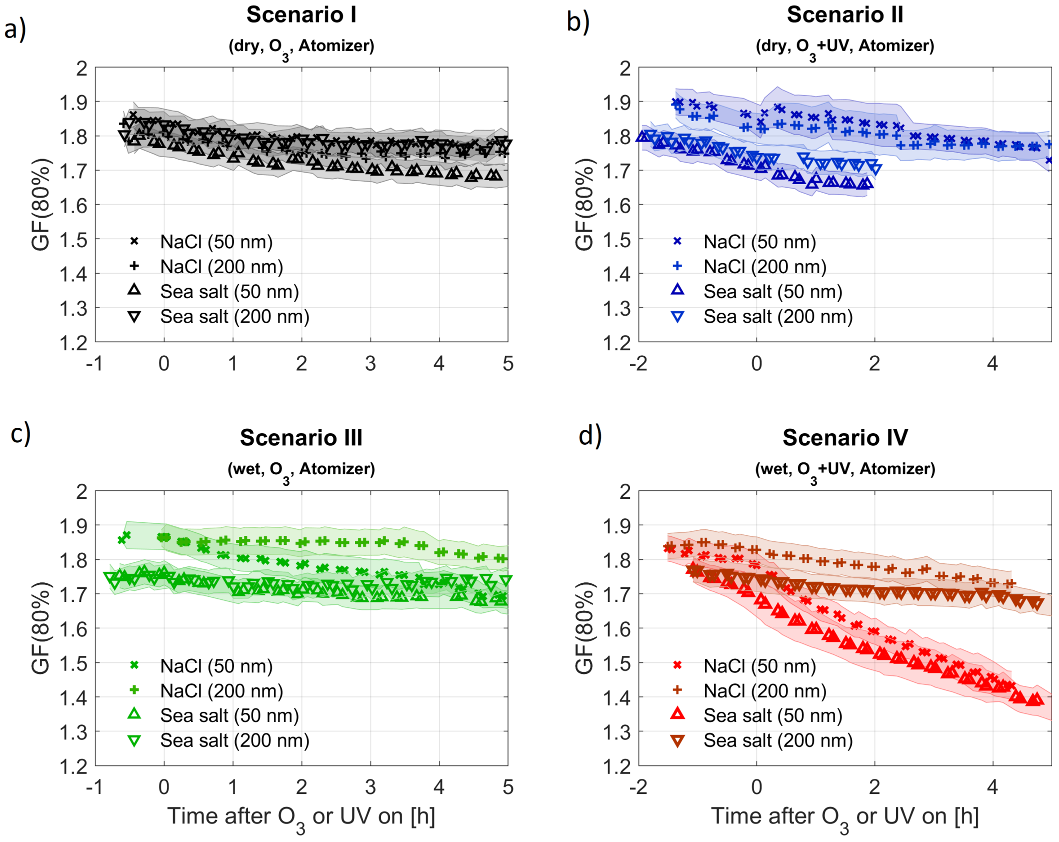

Figure S1: Experiments with Atomiser: Growth factor (GF) measured at RH=80% for NaCl and sea salt for the two different sizes of D_{dry}=50 nm and 200 nm. a) Scenarios I; b) Scenario II; c) Scenario III; d) Scenario IV. The 0-time-point marks the exposure start time to O_3 or OH. During t<0, no oxidant was present in scenarios I and III, while particles together with O_3 were present in the scenarios II and IV.

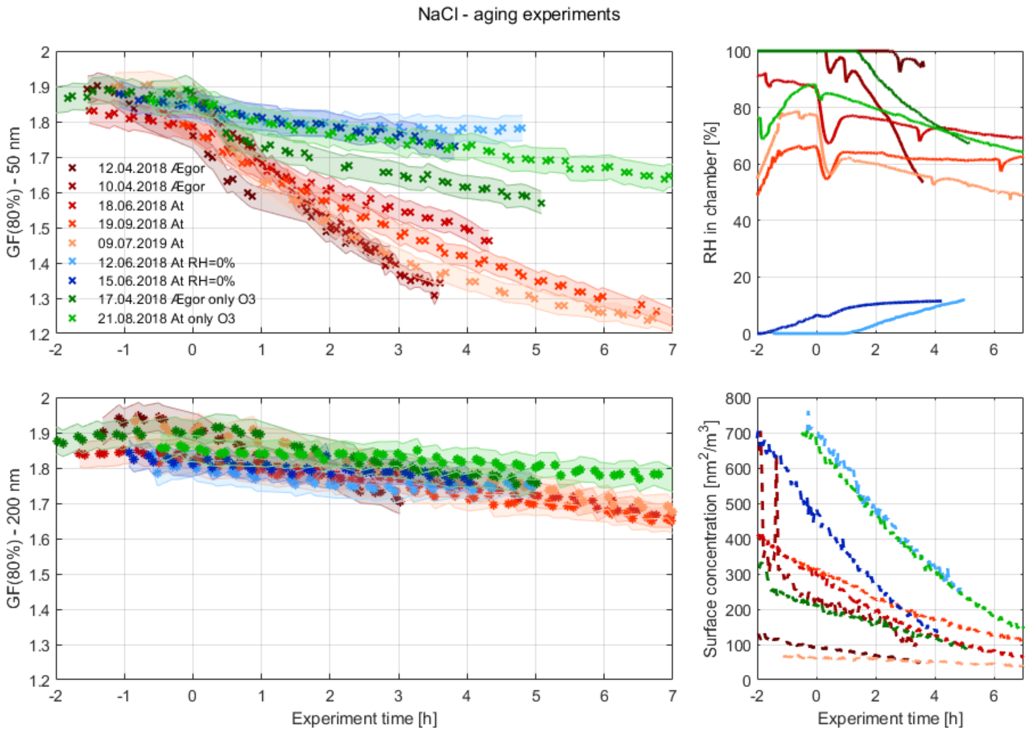

Figure S2: NaCl experiments: GF(80%) for D_{dry}=50 nm and 200 nm are shown for each experiment in each of the scenarios, where "At" stands for atomiser and "Ægor" for experiments using the sea spray simulation tank. The experiments illustrate ageing by exposure to O_3 and UV lights, except where otherwise stated in the legend. Additionally, the RH in the chamber and the surface concentration in the chamber are illustrated.

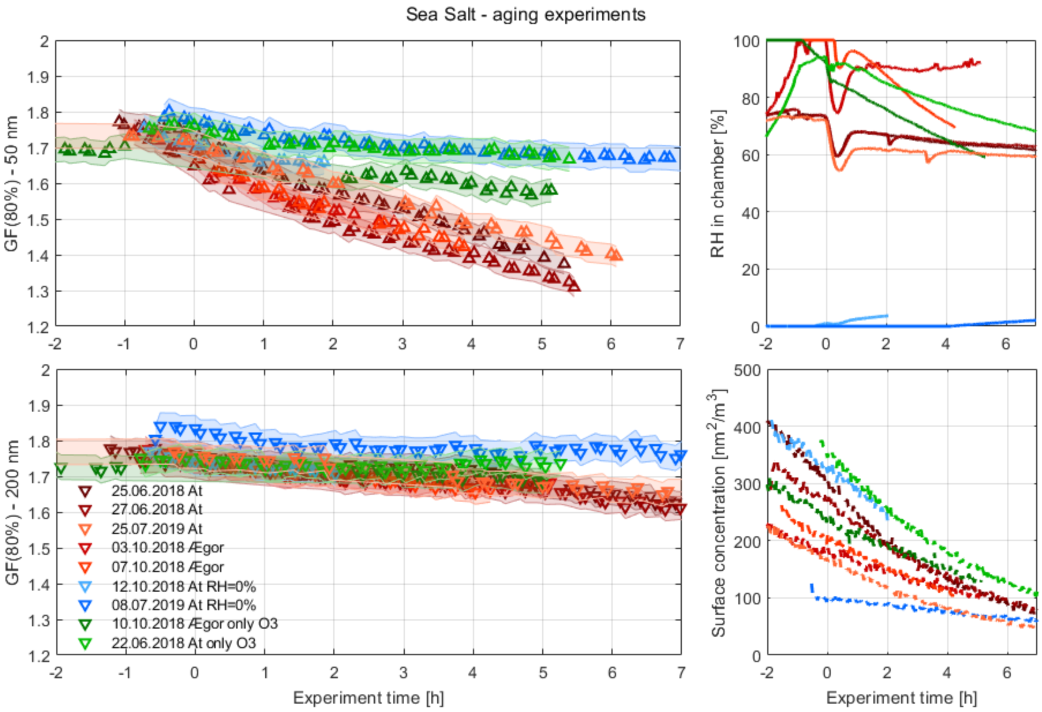

Figure S3: Sea salt experiments: GF(80%) for D_{dry}=50 nm and 200 nm are shown for each experiment in each of the scenarios, where "At" stands for atomiser and "Ægor" for experiments using the sea spray simulation tank. The experiments illustrate ageing by exposure to O_3 and UV lights, except where otherwise stated in the legend. Additionally, the RH in the chamber and the surface concentration in the chamber are illustrated.

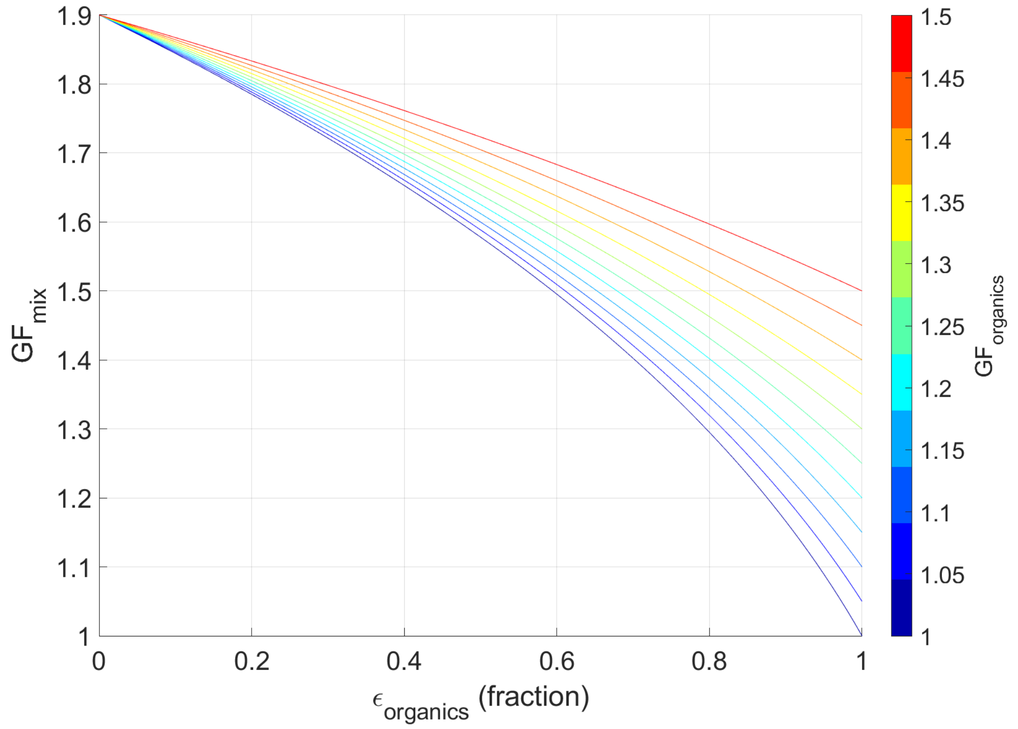

Figure S4: Calculated GF of mixed particles (NaCl, organic) versus volume fraction of the organic compound. Each curve corresponds to an organic with a certain hygroscopicity. The hygroscopicity of the organic is specified by the growth factor ($GF_{organics}$) of a particle consisting only of the given organic compound. Calculations were performed, using a volume weighted mixing rule (ZSR), of the mixed hygroscopic growth factor (GF) assuming a $GF_{inorganic}$ of 1.9 (NaCl) at RH=80% and a varying $GF_{organic}$ from 1-1.5 (colour code). The x-axis denotes the organic volume fraction ($\varepsilon_{organic}$) while the y-axis shows the mixed GF (GF_{mix}).

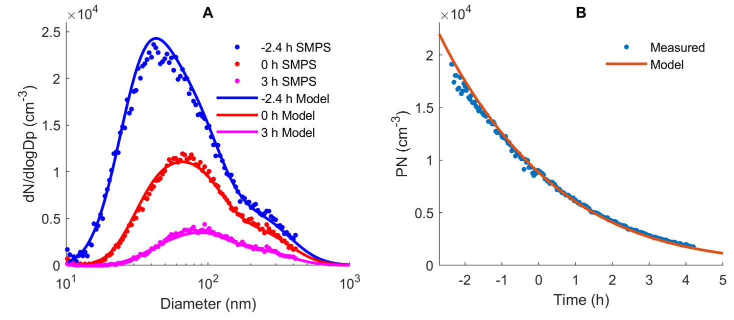

Figure S5: ADCHAM Exp. 7: Panel A: Particle number size distributions at different times of the experiment. Markers show experimental data while lines show modelled data. Panel B: Particle number concentration (PN) as a function of experimental time from experimental and modelled data.

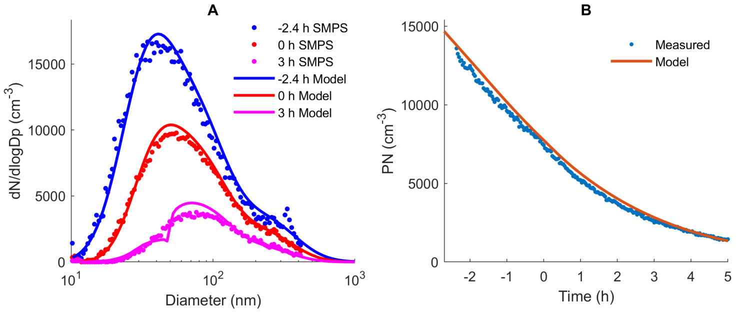

Figure S6: ADCHAM Exp. 8: Panel A: Particle number size distributions at different times of the experiment. Markers show experimental data while lines show modelled data. Panel B: Particle number concentration (PN) as a function of experimental time from experimental and modelled data.

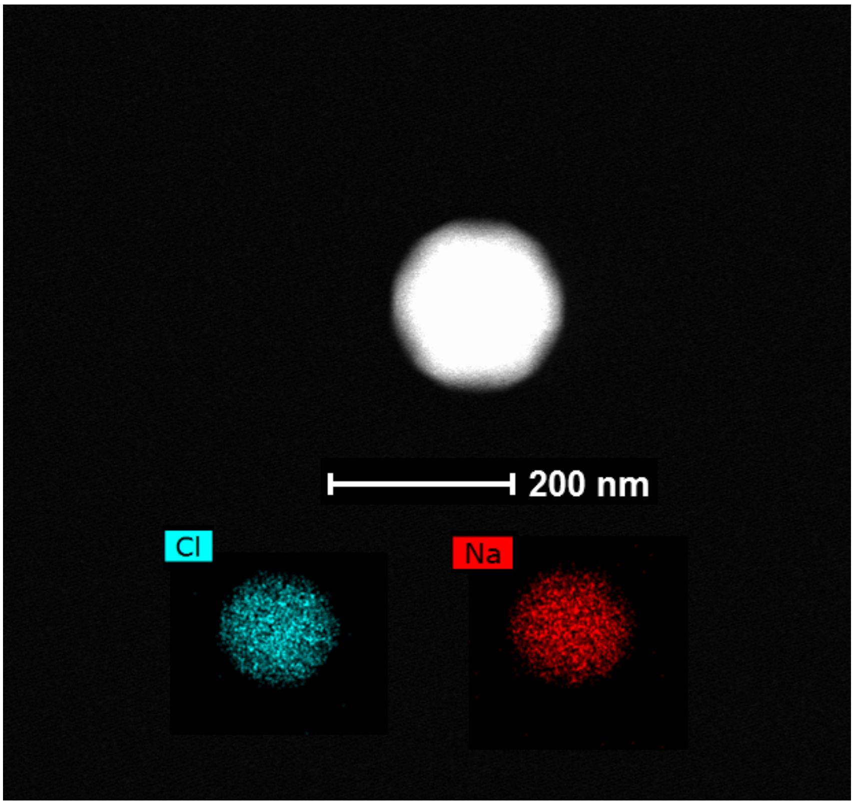

Figure S7: Microscopy (STEM) picture of aged NaCl particle after treatment with a thermodenuder (set at 300°C). Only Na and Cl were found inside this particle. See main manuscript for more information.

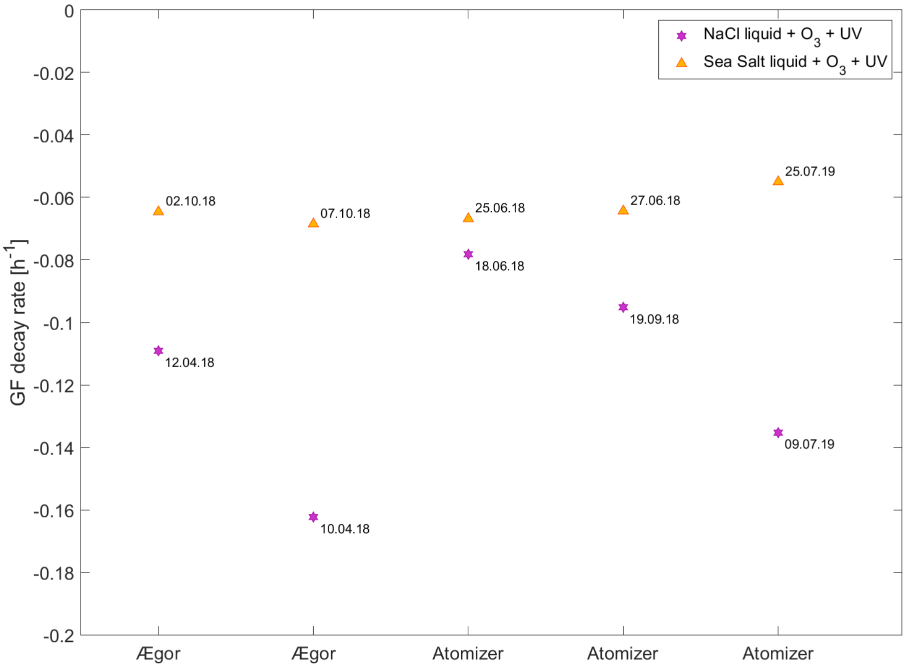

Figure S8: Growth factor decay rates for NaCl and sea salt particles with D_{dry}=50 nm. The values are listed in Table 1.

ADCHAM model simulations:

In order to investigate the processes behind the observed particle number size distribution evolution during the AURA experiments we setup the Aerosol Dynamics gas- and particle-phase chemistry model for laboratory CHAMber studies (ADCHAM)[1;2]. The model was setup for one dry (Exp. 7) and one humid (Exp. 8) experiment with initially pure NaCl particles aged in the presence of O_3 and UV light. In the humid experiments (RH ranging between 68 % and 92 %) the salt particles well assumed to be well mixed liquid water droplets. In the dry experiments (RH ranging between 0 % and 12 %) the particles were instead assumed to be solid crystalline salt particles.The initial NO_x concentration was estimated to be 1 ppb_v based on the NO_x observations in AURA. The measured temperature and RH time series were used as input to the model. The only condensable/evaporating species considered in the model simulations were HCl, HNO_3, H_2SO_4 and NH_3. The modelled particle number size distribution was represented by 100 fixed size bins in the size range 10 nm to 1.37 μm in diameter. The initial particles were assumed to be composed of pure NaCl. The initial particle number size distribution were estimated based on the observed particle number size distribution from the SMPS. The particle wall losses were modelled considering the particle wall deposition of charged and non-charged particles using the particle wall loss parameterizations described in Roldin et al[1]. The key unknown parameters in these parameterizations are the electric field strength inside the chamber and the friction velocity. For the simulations performed in this work we used an electric field strength of 1.0 V/cm and a friction velocity of 0.03 m/s.

The model also simulated the gradual dilution of the smog chamber because of the instrument sampling. The total instrument sampling rate (q) was 15 l/min. With this rate the chamber bag volume, which initially is 5 m^3, should be completely compressed (0 m^3) after 5.5 hours. However, based on observations of the bag, we know that this is not the case and instead sampling from the bag was possible for much longer. This can only happen if a fraction of air outside the bag can leak into the chamber. From observations of the bag we estimate that the chamber volume can be compressed from initially 5 m^3 to a minimum of 3 m^3 within the time frame of the experiments. In order to simulate this in the model we introduce the variable $f_{compaction}$ that describes the fraction of the sampled air which results in additional compaction of the modelled chamber volume, where $f_{compaction} = 1$ means that the bag is not compressed at all and $f_{compaction} = 0$ that the bag has already been compressed to its minimum. If the chamber volume at time (t) is denoted as V(t), the estimated minimum

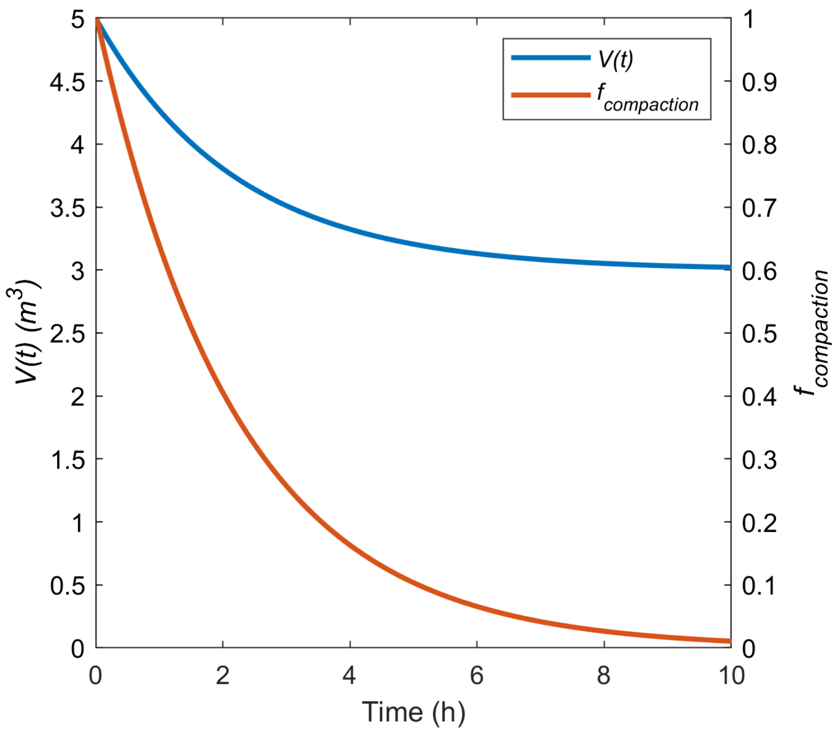

Figure S9: Chamber volume at time (t) (V(t)) and $f_{compaction}$ as a function of time as calculated using Eq. 1. and Eq. 2.

chamber volume as V_{min} and the initial volume of the chamber as V_0 we can formulate a simplified parametrization for $f_{compaction}$:

$$f_{\mathrm{compaction}} = (V_t - V_{\mathrm{min}})/(V_0 - V_{\mathrm{min}}) \tag{1}$$

and express the change in chamber volume from one model time step (Δt = 5 s) to the next as:

$$V_{t+\Delta t} = V_t - f_{compaction} q \Delta t \tag{2}$$

and then considering the change in the number density (concentration, c) of all species (e.g. particles) due to the dilution within one time step as:

$$c_{t+\Delta t} = c_t (V_t - q\Delta t)/V_{t+\Delta t} \tag{3}$$

The results from Eq. 1. and 2 are illustrated in Fig. S9. The remaining fraction of the sampled air ($1 - f_{\mathrm{compaction}}$) was assumed to be particle free air leaking into the chamber from outside, which resulted in a gradual dilution of the chamber volume and decreasing aerosol particle concentration.

Figure S5- S6 shows the modelled and observed particle number size distributions and total particle number concentration for Exp. 7 and 8. The close agreement between the modelled and observed particle number size distribution and total particle number concentrations

during both experiments show that the particle number size distribution and particle number concentration evolution during the AURA experiments are mainly governed by coagulation, particle wall losses and gradual dilution of the chamber because of the instrument sampling which was 15 l/min.

Bibliography

[1] P. Roldin, A. C. Eriksson, E. Z. Nordin, E. Hermansson, D. Mogensen, A. Rusanen, M. Boy, E. Swietlicki, B. Svenningsson, A. Zelenyuk, and J. Pagels. Modelling non-equilibrium secondary organic aerosol formation and evaporation with the aerosol dynamics, gas- and particle-phase chemistry kinetic multilayer model adcham. *Atmospheric Chemistry and Physics*, 14(15):7953–7993, 2014. doi: 10.5194/acp-14-7953-2014. URL `https://www.atmos-chem-phys.net/14/7953/2014/`.

[2] P. Roldin, E. Mikael, T. Kurtén, T. Olenius, M. P. Rissanen, N. Sarnela, J. Elm, P. Rantala, L. Hao, N. Hyttinen, L. Heikkinen, D. R. Worsnop, L. Pichelstorfer, C. Xavier, P. Clusius, E. Öström, T. Petäjö, M. Kulmala, H. Vehkamäki, A. Virtanen, I. Riipinen, and M. Boy. The role of highly oxygenated organic molecules in the boreal aerosol-cloud-climate system. *Nature Communications*, 10:4370, 2019. doi: https://doi.org/10.1038/s41467-019-12338-8.
